# Alterations in Circulating Immune Cells in Neovascular Age-Related Macular Degeneration

**DOI:** 10.1038/srep16754

**Published:** 2015-11-17

**Authors:** Judith Lechner, Mei Chen, Ruth E. Hogg, Levente Toth, Giuliana Silvestri, Usha Chakravarthy, Heping Xu

**Affiliations:** 1 Centre for Experimental Medicine, School of Medicine, Dentistry and Biomedical Sciences, Queen’s University Belfast, Belfast, UK

## Abstract

Neovascular age-related macular degeneration (nAMD) is the leading cause of irreversible blindness in developed countries. Recent advances have highlighted the essential role of inflammation in the development of the disease. In addition to local retinal chronic inflammatory response, systemic immune alterations have also been observed in AMD patients. In this study we investigated the association between the frequency of circulating leukocyte populations and the prevalence as well as clinical presentations of nAMD. Leukocyte subsets of 103 nAMD patients (most of them were receiving anti-VEGF therapy prior to enrolment) and 26 controls were analysed by flow cytometry by relative cell size, granularity and surface markers. Circulating CD11b^+^ cells and CD16^hi^HLA-DR^−^ neutrophils were significantly increased (*P *= 0.015 and 0.009 respectively) in nAMD when compared to controls. The percentage of circulating CD4^+^ T-cells was reduced in nAMD patients without subretinal fibrosis (*P *= 0.026) compared to patients with subretinal fibrosis. There was no correlation between the percentage of circulating leukocytes and the responsiveness to anti-VEGF therapy in nAMD patients. Our results suggest that higher levels of circulating CD11b^+^ cells and neutrophils are associated with nAMD and that reduced levels of CD4^+^ T-cells are associated with the absence of subretinal fibrosis in nAMD.

Age related macular degeneration (AMD) is the leading cause of blindness in the developed world in individuals above the age of 55^1^,^2^. Visual acuity is usually unaffected in early stages of the disease, which is characterised by the presence of drusen which are deposits that occur in the outer retina and Bruch’s membrane-retinal pigment epithelial complex^3^. Visual loss occurs when there is degeneration and loss of photoreceptors, retinal pigment epithelium (RPE), and choriocapillaries (atrophic AMD) or when neovascularisation supervenes (neovascular AMD (nAMD)). When abnormal neovascular complexes are present under the retinal pigment epithelium this is referred to as type 1 choroidal neovascularisation (CNV). When these complexes invade the subretinal space these are referred to as type 2 membranes^4^. Neovascular complexes are also thought to arise *de novo* within the retina known as retinal angiomatous proliferation (RAP) and sometimes referred to as type 3 membranes. The neovascular lesions in RAP can fuse with choroidal new vessels (i.e., CNV)^5^.

Apart from aging, a plethora of risk factors have been shown to contribute to the development of AMD^6^,^7^. However, the pathogenesis of AMD remains incompletely understood. Compelling evidence suggests that inflammation plays a critical role in the aetiology of AMD^8^,^9^, and molecular dissection of immunological pathways to identify critical events will offer opportunities for the development of novel therapies. Genetic studies have identified an association of AMD risk with immune related genes, including various complement related genes (CFH^10^, C3^11^, C2 and CFB^12^) and chemokine receptor CX3CR1^13^. Complement components and other inflammatory molecules have been found in drusen of AMD patients^14^ and histological studies have detected macrophages in the vicinity of drusen^15^ as well as in retinal lesions^16^ in eyes from AMD patients, suggesting a local chronic inflammatory response in AMD.

In addition to the local inflammatory response, systemic immune alterations have also been observed in AMD patients. Increased serum levels of complement components^17^ and pro-inflammatory cytokines such as IL-1α, IL-1β and IL-17^18^ have been reported in patients with AMD. Changes in the expression of chemokine receptors (e.g. CCR2 and CX3CR1)^19^,^20^, complement regulatory proteins (e.g. CD46, CD59)^21^ and CD200^22^ in circulating monocytes and lymphocytes have been observed in AMD patients. In addition, increased total white blood cell count was found to be associated with early AMD development^23^. More recently, a study has reported increased neutrophil/lymphocyte ratio in AMD patients compared to controls^24^. The involvement of systemic immune activation in AMD pathogenesis was further supported by a study from Nussenblatt *et al*.^25^ which showed that systemic immune suppression could alter the clinical course of nAMD and in some cases could reduce the monthly rate of intravitreal anti-VEGF injections to control disease progression when compared to pre-study injection rates or to injection rate in the control group. However, due to lack of knowledge in the detailed immune pathways involved in nAMD, more effective immune therapy has yet to be developed.

This research was performed to fully characterise the immunophenotype of nAMD and to understand whether a specific immunophenotype is related to certain clinical presentations or the responsiveness to anti-VEGF therapy in nAMD patients.

## Results

### General Information of Human Participants

In total, 129 participants were included in this study consisting of 26 controls and 103 nAMD patients. No significant differences were found between nAMD patients and controls regarding age, gender, family history of AMD, history of cardiovascular disease, history of hypertension, history of diabetes and BMI. The smoking habits were similar in both groups as well as the number of patients taking cardiovascular medication, vitamins and low dose aspirin (Table 1).

**Figure 1:**
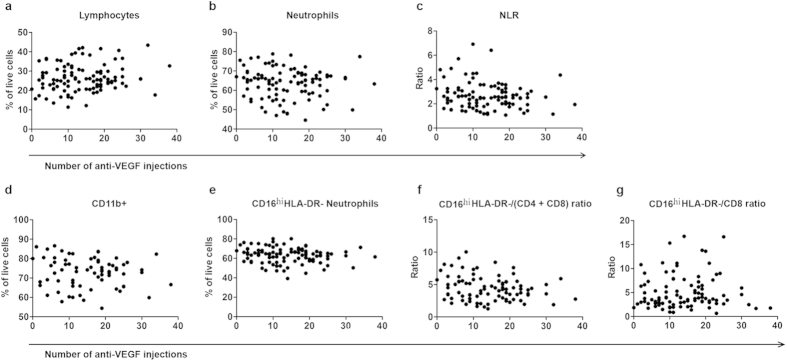
The relationship between the number of anti-VEGF injections and different subsets of circulating immune cells. The number of anti-VEGF injections received by nAMD patients did not correlate with the percentage of lymphocytes **(a)**, neutrophils **(b)**, the NLR **(c)**, with the percentage of CD11b^+^ cells **(d)**, CD16^hi^HLA-DR^−^ neutrophils **(e)**, the CD16^hi^HLA-DR^−^/(CD4 + CD8) ratio **(f)** or the CD16^hi^HLA-DR^−^/CD8 ratio **(g)**.

Most of the nAMD participants were receiving anti-VEGF therapy prior to enrolment and the average number of anti-VEGF injections received prior to blood collection was 14.3 ± 8.7 (range 0–38). No participant had received anti-VEGF treatment within 4 weeks prior to blood collection. There was no correlation between the number of anti-VEGF injections received and the percentage of lymphocytes (R^2^* *= 0.16; *P *= 0.105, Fig. 1a), neutrophils (R^2^* *= −0.09; *P *= 0.376, Fig. 1b), neutrophil/lymphocyte ratio (NLR) (R^2^* *= −0.15; *P *= 0.137, Fig. 1c), CD11b^+^ cells (R^2^* *= −0.11; *P *= 0.370, Fig. 1d), CD16^hi^HLA-DR^−^ neutrophils (R^2^* *= −0.15; *P *= 0.136, Fig. 1e), CD16^hi^HLA-DR^−^/(CD4 + CD8) ratio (R^2^* *= −0.17; *P *= 0.105, Fig. 1f) and CD16^hi^HLA-DR^−^/CD8 ratio (R^2^* *= −0.07; *P *= 0.490, Fig. 1g).

### Leukocyte Subsets in nAMD Patients and Controls

**Figure 2:**
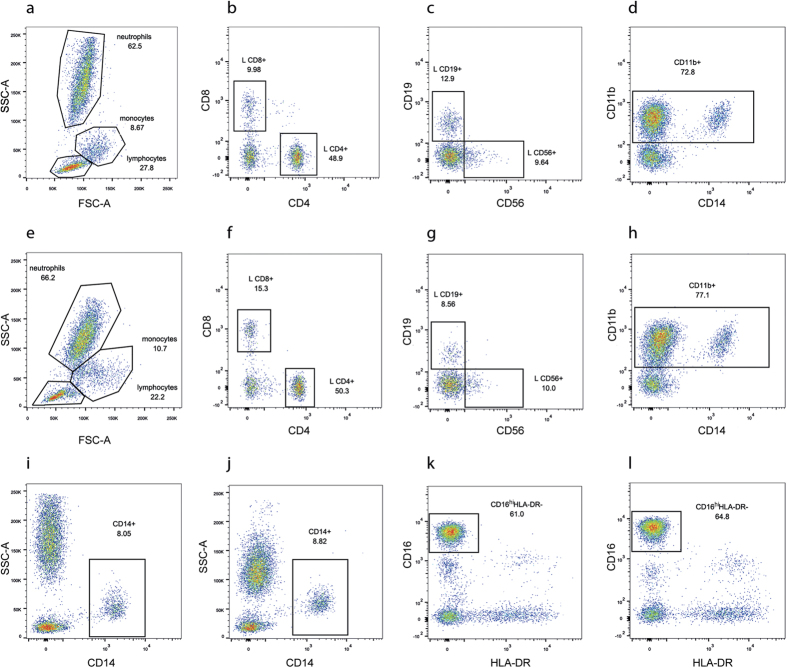
Gating strategy used in flow cytometry analysis to detect different subsets of circulating immune cells. Sample data from a control (**a**–**d**,**i**,**k**) and a nAMD patient (**e**–**h**,**j**,l) are shown. Lymphocyte, monocyte and neutrophil populations were gated on a forward scatter (FSC)/side scatter (SSC) plot (**a**,**e**). Lymphocytes were then further gated to determine CD4^+^, CD8^+^ (**b,f**), CD19^+^ and CD56^+^ lymphocytes (**c**,**g**). Live cells were further gated to determine CD11b^+^ cells (**d**,**h**), CD14^+^ monocytes (**i,j)** and CD16^hi^HLA-DR^−^ neutrophils (**k**,**l**).

White blood cells (leukocytes) can be divided into lymphocytes, monocytes and neutrophils based on their cell size and granularity using FSC/SSC plot in flow cytometry (Fig. 2a,e). The percentage of lymphocytes was significantly decreased in nAMD patients compared to age-matched healthy controls in both the univariate and multivariate analysis (Table 2). Neutrophils were significantly increased in the univariate analysis (*P *= 0.025), however, after taking age and gender into account there was only a tendency of increased neutrophil percentage (*P *= 0.052) in nAMD compared to controls (Table 2). In addition, the NLR was significantly increased in the nAMD group compared to the control group in both univariate and multivariate anlaysis (Table 2). There was no association between the percentage of lymphocytes, neutrophils or NLR and other confounders.

After the initial analysis, leukocyte subsets were further analysed using specific cell surface antigens, including CD4^+^ and CD8^+^ T-cells, CD19^+^ B-cells, CD56^+^ natural killer cells, CD11b^+^ myeloid cells, CD14^+^ monocytes and CD16^hi^HLA-DR^−^ neutrophils (Fig. 2a–l). We detected two populations of CD16^+^HLA-DR^−^ cells, CD16^hi^HLA-DR^−^ and CD16^lo^HLA-DR^−^ (Fig. 2k,j). Back-gating of these two populations on a FSC/SSC plot showed that all the CD16^hi^HLA-DR^−^ cells had a FSC/SSC profile of neutrophils (Supplemental Figure S1a, S1b), whereas the CD16^lo^HLA-DR^−^ cells had a FSC/SSC profile of lymphocytes (Supplemental Figure S1a, S1d). Furthermore, both CD16^hi^HLA-DR^−^ and CD16^lo^HLA-DR^−^ cells were negative for CD14 (Supplemental Figure S1c, S1e). The results suggest that the CD16^hi^HLA-DR^−^ cells are neutrophils and the CD16^lo^HLA-DR^−^ cells are likely nature killer cells^26^.

The percentage of CD11b^+^ cells was significantly increased in the univariate and multivariate analysis as well as the percentage of CD16^hi^HLA-DR^−^ neutrophils in nAMD patients compared to controls (Table 2). There was a significant increase in the CD16^hi^HLA-DR^−^/(CD4 + CD8) ratio in the nAMD group in the univariate analysis (*P *= 0.031). On adjustment for age and gender, statistical significance was lost (Table 2). There was no association between the percentage of CD11b^+^ cells, CD16^hi^HLA-DR^−^ neutrophils or CD16^hi^HLA-DR^−^/(CD4 + CD8) ratio and other confounders.

There was a tendency of increased CD16^hi^HLA-DR^−^/CD8 ratio in nAMD patients compared to controls in the univariate and multivariate analysis adjusting for age and gender (Table 2, *P *= 0.051 and 0.072 respectively). The CD16^hi^HLA-DR^−^/CD8 ratio was also significantly associated with history of cardiovascular disease and low-dose aspirin intake. Interestingly, when history of cardiovascular disease and low-dose aspirin intake were added to the multivariable logistic regression analysis, the increase in the CD16^hi^HLA-DR^−^/CD8 ratio in nAMD patients compared to controls was found to be significant (*P *= 0.041) (Table 2).

The average percentages of leukocyte sub-populations in control subjects in our study were similar to those reported previously^27^: lymphocytes: 30.7 ± 9.3% compared to 30.45 ± 7.8% in our study; monocytes: 8.9 ± 3.1% compared to 7.28 ± 1.88% in our study; CD16^+^ neutrophils: 60.0 ± 25.3% compared to 57.8 ± 9.1% CD16^hi^HLA-DR^−^ neutrophils in our study; CD19^+^ B-cells: 4.1 ± 2.0% compared to 3.4 ± 2.16% in our study; T-cells and NK cells: 22.7 ± 6.7% compared to 21.3 ± 8.5% (CD4^+^, CD8^+^ and CD56^+^ cells) in our study.

### Leukocyte Subsets and Responsiveness to Anti-VEGF Therapy

Of the 103 nAMD patients, 48 (47%) responded completely to the anti-VEGF therapy, 52 (50%) partially responded to the therapy and 3 patients (3%) did not respond to the therapy. Due to the limited number of non-responders in this study, this group was not included in the statistical analysis. No differences in leukocyte subsets were identified when comparing complete responders with partial responders (Supplemental Table S1).

### Leukocyte Subsets and Subretinal Fibrosis

Among nAMD patients, subretinal fibrosis was present in 37 patients (36%). The level of CD4^+^ T-cells was significantly lower in patients without fibrosis (10.82%) than in patients with fibrosis (12.44%) in both the univariate and multivariate analysis (*P *= 0.026) (Table 3). The level of CD4^+^ T-cells was similar in nAMD patients with fibrosis and age-matched healthy controls (Table 3). The percentage of CD4^+^ T-cells was not associated with any other confounders. There was no difference in the level of other leukocyte subsets between patients with and without subretinal fibrosis (Table 3).

### Leukocyte Subsets in Patients with CNV and RAP

Details regarding the type of neovascularisation were available for 79 out of 103 nAMD patients. Of those, 66 (84%) were diagnosed with CNV and 13 (16%) were diagnosed with RAP. No significant differences were found in leukocyte subsets between CNV and RAP, although there was a tendency of increased percentage of CD11b^+^ cells in RAP patients compared to CNV patients (76.72% and 72.00% respectively; *P *= 0.082) (Table 4).

When the two groups were compared to the control group, RAP nAMD patients had a significantly increased percentage of CD11b^+^ cells in the univariate and multivariate analysis (*P *= 0.009 and 0.033 respectively, Table 4). Patients with CNV had a tendency of increased percentage of CD11b^+^ cells in the univariate analysis (*P *= 0.086) and this p-value was found to be significant when age and gender were taken into account in the multivariate analysis (*P *= 0.046). Additionally, CNV patients had a significantly increased percentage of CD16^hi^HLA-DR^−^ neutrophils when compared to controls in both the univariate and multivariate analysis (*P *= 0.027 and 0.015 respectively). While patients with RAP had the highest CD16^hi^HLA-DR^−^ neutrophil percentage, the difference was not statistically significant when compared to controls (Table 4). The percentage of lymphocytes was lowest in RAP patients but the reduction did not reach statistical significance when compared to controls (*P *= 0.064) (Table 4). There were no differences between CNV, RAP and controls in other leukocyte populations or neutrophil/lymphocyte ratios (Table 4).

Details regarding the type of CNV were available for 58 out of 66 CNV patients. Seven patients (12%) were diagnosed with classic CNV, 32 (55%) with occult CNV and 19 (33%) with a mixed phenotype. There were no differences in any of the leukocyte subsets between the three groups of CNV (Supplemental Table S2).

## Discussion

Recent findings suggest that systemic immune alterations may play a role in the pathogenesis of AMD. In addition to increased levels of complement components and pro-inflammatory cytokines in the serum of AMD patients^17^,^18^, changes in leukocytes, especially monocytes have been reported in AMD^20^,^21^,^22^. In this study, we found that the percentages of circulating CD11b^+^ cells and CD16^hi^HLA-DR^−^ neutrophils were significantly increased in nAMD patients when compared to controls. We further found a higher percentage of CD4^+^ T-cells in patients with subretinal fibrosis when compared to patients without subretinal fibrosis. We found no correlation between the percentage of circulating leukocytes and the responsiveness to anti-VEGF therapy in nAMD patients. Our observation may have important implications in future management of nAMD.

The leukocyte antigen CD11b is expressed on both neutrophils and monocytes. There was no significant difference in the percentage of CD14^+^ monocytes between nAMD patients and controls indicating that the increased percentage of CD11b^+^ cells is due to the increased percentage of CD16^hi^HLA-DR^−^ neutrophils. Increased neutrophil/lymphocyte ratio in AMD patients has been observed by others^24^. The precise role of neutrophils in AMD pathogenesis remains elusive. Neutrophils are the most abundant leukocytes in the blood and respond rapidly to inflammatory and infectious stimuli. Traditionally, neutrophils were considered as innate immune cells that engulf bacteria and phagocytose apoptotic cells during acute inflammation^28^. In recent years it has become evident that the complexity of neutrophil functions is far more widespread than previously thought and that they play an active role in many aspects of the innate immune response^29^. Activated neutrophils can produce pro-inflammatory cytokines such as IL-8 and IL-1β, which can further recruit other immune cells^30^,^31^. Neutrophils can also stimulate T-cell activation through expression of MHC class II^32^,^33^. Indeed, neutrophils have been shown to promote inflammation in a number of human inflammatory diseases such as arthritis^34^ and atherosclerosis^35^. There is now increasing evidence that neutrophils also play an active role in angiogenesis^36^,^37^. Activated neutrophils produce proteases, in particular matrix metalloproteinase-9 (MMP-9), which degrade and remodel the extracellular matrix, a crucial process in angiogenesis^38^. Neutrophils are known to be involved in retinal angiogenesis in laser-induced CNV^39^, probably through disruption of the RPE barrier integrity by releasing MMP-9^40^. In addition, neutrophils can promote angiogenesis by releasing pro-angiogenic factors such as VEGF and IL-8^41^. Furthermore, VEGF can recruit pro-angiogenic neutrophils that produce high levels of MMP-9^37^.

Subretinal fibrosis develops in some patients with late stage nAMD as a result of subretinal haemorrhages and can cause irreversible central vision loss^4^. It is unknown why some patients develop subretinal fibrosis while others do not. In the present study we report that patients without subretinal fibrosis have a lower percentage of CD4^+^ T-lymphocytes (10.82%) when compared to patients who developed subretinal fibrosis (12.44%). Interestingly, patients who developed subretinal fibrosis had similar levels of CD4^+^ T-cells as controls (12.21%) suggesting lower levels of CD4 might reduce the risk of developing subretinal fibrosis. While the difference between patients with and without subretinal fibrosis was statistically significant (*P *= 0.026), the clinical relevance of such a small difference (1.6%) warrants further investigation. CD4^+^ T-cells have previously been linked to the formation of fibrosis in other tissues in human diseases and in animal models. Increased numbers of CD4^+^ cells have been found in the lungs of patients with lung fibrosis^42^, and in fibrotic kidneys of patients with IgA nephropathy^43^. Neutralisation of CD4^+^ T-cells decreased the formation of fibrosis in a mouse model of tail lymphedema^44^ and reduced the intensity of silica induced lung fibrosis^45^, although, other studies reported controversial results^46^. Recent discoveries have shown that different subsets of CD4^+^ cells have different roles in fibrosis with some subpopulations (Th17, Th2) displaying more pro-fibrotic properties while others (Th22) have more anti-fibrotic properties or a dual role with pro- and anti-fibrotic functions (Th9, Tregs)^47^. Th17 is reported to be associated with AMD^48^. Our results suggest that CD4 T-cells might be associated with the development of subretinal fibrosis, however further studies including larger number of patients are required to confirm those findings.

Neovascular AMD is currently treated with regular intravitreal injections of anti-VEGF agents, including ranibizumab (Lucentis, Genentech, San Francisco, CA), bevacizumab (Avastin, Roche, Basel, Switzerland) and more recently aflibercept (Eylea, Regeneron, Tarrytown, New York). However, up to 10% of patients do not respond to the treatment and approximately half of the patients only partially respond to the therapy^49^. As leukocytes are known to play important roles in nAMD and different leukocytes may produce different types of angiogenic growth factors (in addition to VEGF), we hypothesised that alterations in leukocyte subsets might be associated with response to anti-VEGF treatment. We found no differences in leukocyte levels between patients completely or partially responding to anti-VEGF therapy, suggesting that immunophenotype is unlikely associated with the response to anti-VEGF therapy. However, additional studies including large numbers of patients are necessary to confirm those results. Predictors of treatment outcomes would be beneficial in performing the most appropriate treatment in the most cost effective way for each patient and would allow giving more accurate prognosis. A number of studies have searched for predictors of treatment response by looking at clinical and genetic factors^49^. However, a strong marker has not yet been identified thus far.

In this study, we found a significantly increased percentage of CD11b^+^ cells in both CNV patients and RAP patients, whereas the percentage of CD16^hi^HLA-DR^−^ neutrophils was only increased statistically in patients with CNV but not RAP when compared to controls. However due to the small number of participants in the RAP group (n* *= 13), these results must be interpreted with caution. Further studies using larger patient samples are necessary to see whether alterations in circulating immune cells are related to a specific type of neovascularisation in nAMD.

There are a number of limitations in the current study. Firstly, there was a small difference in age between nAMD patients and controls although this was not statistically significant. The median age was higher in the nAMD group compared to controls (79.5 and 76.7 years respectively). Secondly patients were recruited at different times following diagnosis of nAMD. Consequently, some patients enrolled at an early stage of nAMD and classified as having no fibrosis, might still develop fibrosis during the course of the disease. Subdividing the nAMD group into CNV/RAP, fibrosis absent/present and complete/partial responders was carried out as a secondary analysis and the study was not planned specifically to detect these associations (power < 80%), we recognise that the conclusions drawn from this part of the study are tentative and will require confirmation in a larger study. There are four times more cases enrolled in the study than controls which is the maximum imbalance acceptable for case-control studies and might affect the statistical analyses. Finally, patients enrolled in this study were receiving anti-VEGF treatment prior to enrolment which may have altered circulating immune cell levels although there was no correlation with the number of anti-VEGF injections received and the percentage of immune cells. Furthermore the drug in use in our study site was ranibizumab which is cleared more rapidly from the systemic circulation and has the least effect on serum VEGF levels when compared to other anti-VEGF agents^50^. Despite the limitations, the present study has uncovered novel systemic immune alterations in nAMD patients and might have important implications in the management of nAMD.

In summary, we show that neutrophils were increased in patients with nAMD suggesting increased systemic innate immune activation in nAMD patients. Our results also indicate that CD4^+^ T-cells may be related to the development of subretinal fibrosis in nAMD patients. Further studies about the role of neutrophils in nAMD and the role of CD4 T cells in subretinal fibrosis may advance our understanding in the immunopathogenesis of nAMD and uncover novel targets for therapy.

## Methods

### Study Participants

The study protocol was approved by the Research Ethics Committee of Queen’s University Belfast and Belfast Health and Social Care Trust Research Ethics Committee, UK. The study procedures were performed in accordance with the tenets of the Declaration of Helsinki. Written informed consent was obtained from every participant.

All participants were recruited from the macular disease clinics in Belfast (Belfast Health and Social Care Trust, UK). Relatives and spouses, who attended the clinic with patients and who were without retinal disease (confirmed by fundus photography and optical coherence tomography (OCT)) were recruited as controls. All participants were above 53 years of age and structured questionnaires were used to ascertain a history of medical conditions, current medication, family history of AMD, smoking habits (current, ex-smoker, never smoker) and BMI. Patients with systemic inflammatory or autoimmune disease were excluded from the study as well as patients receiving steroid drug treatment. The diagnosis of nAMD was confirmed by an experienced ophthalmologist on clinical examination using fundus photography, fluorescein angiography and OCT. In this study most of the participants were receiving anti-VEGF therapy prior to enrolment. The number of anti-VEGF (ranibizumab, trade name Lucentis, Genentech, San Francisco, CA) injections received by each patient prior to blood collection was ascertained from the medical records. Peripheral blood samples were drawn in tubes containing ethylenediaminetetraacetic acid (EDTA) as anticoagulant between 9:00 and 12:00 am and processed within 3 hours of collection. Responsiveness to treatment was defined based on the participant achieving a fluid free macula at any stage during follow up. In addition the status of whether a patient was fluid free at the month 3 and month 6 examinations was also recorded. Participants were classified into the following 3 categories: Complete responder: Resolution of leakage at any point in time during follow up; Partial responder: Exhibiting dependence of VEGF inhibitors but a fluid free macula was never achieved; Non responder: No morphological improvement or worsening.

### Flow Cytometry of Human Blood Samples

Freshly drawn blood samples (30 μl) were incubated with fluorochrome-labelled antibodies in a total volume of 100 μl FACS buffer (PBS/1% fetal calf serum (FCS)) for 45 min in the dark at 4 °C. Red blood cells were lysed by incubating the sample with 2 ml of 1X red blood cell lysis solution (BD Biosciences, Oxford, UK) for 10 min at room temperature (RT). After thorough washes samples were fixed in 1% paraformaldehyde (PFA) for 30 min in the dark at 4 °C. Samples were then washed and resuspended in PBS. All samples were examined by flow cytometry (FACS CANTO II; BD Biosciences), and data analysed blindly using the FlowJo software (version 10.07 for Windows, Tree Star, Ashland, OR, USA). The following anti-human antibodies were used for identification of leukocyte subsets: CD14-APC-Cy7, CD19-FITC, CD16-Pacific Blue, CD56-PE, CD8-PE-Cy7, CD4-Pacific Blue, CD11b-APC (all from BD Biosciences, UK) and HLA-DR-PE (eBioscience, UK). Live cells were gated for further analysis of leukocyte subsets. Different subsets of leukocytes were identified by relative cell size and granularity (FSC/SSC) or cell surface markers (Fig. 1). The neutrophil/lymphocyte ratio (NLR) was calculated by dividing the percentage of neutrophils by the percentage of lymphocytes.

### Statistical Analysis

Statistical analysis was performed using the Statistical Package for the Social Sciences, Windows version 21 (SPSS Inc, Armonk, NY). Categorical demographic and clinical data were compared using Pearson’s chi-square test. The distribution of continuous variables was assessed for normality using the Kolmogorov-Smirnov test and transformed if necessary. Normally distributed continuous samples were then compared using the Independent samples t-test or one-way ANOVA. If transformation failed to improve the distribution, Mann-Whitney U test was used for the comparisons. For the associations that were significant in the univariate analysis, multivariable logistic regression, using a generalised linear model, was performed to adjust for age and gender. All variables were also tested for association with family history of AMD, history of cardiovascular disease, history of hypertension, history of diabetes, smoking habits, BMI, taking of cardiovascular medication, vitamins and low dose aspirin using the Independent samples t-test or one-way ANOVA. If significant associations were identified, those confounders were also included in the multivariable logistic regression analysis. Pearson’s correlation was used to assess the correlation between the number of anti-VEGF injections a patient had received and percentage of leukocyte subsets. Data were presented as mean ± standard deviation (SD) calculated from untransformed variables even if the statistical analysis was performed on transformed variables. *P* values <0.05 were considered statistically significant.

## Additional Information

**How to cite this article**: Lechner, J. *et al*. Alterations in Circulating Immune Cells in Neovascular Age-Related Macular Degeneration. *Sci. Rep.* **5**, 16754; doi: 10.1038/srep16754 (2015).

## Supplementary Material

Supplementary Information

## Figures and Tables

**Table 1:** Demographic and clinical characteristics of nAMD patients and controls.

	**All (n*** *= **129)**	**Controls (n*** *= **26)**	**nAMD (n*** *= **103)**	***P*** **value nAMD vs Control**
Age (median (range)), years	79.1 (53–94)	76.7 (59–92)	79.5 (53–94)	0.065^1^
Female sex (number (%))	70 (54)	12 (46)	58 (56)	0.385^2^
Have history of AMD (number (%))	30 (23)	5 (19)	25 (24)	0.796^2^
Have history of cardiovascular disease (number (%))	29 (22)	7 (27)	22 (21)	0.601^2^
Have history of hypertension (number (%))	75 (58)	17 (65)	58 (56)	0.506^2^
Have history of diabetes (number (%))	15 (12)	2 (8)	13 (13)	0.734^2^
Body Mass Index (mean ± SD)	26.4 (4.9)	27.1 (5.6)	26.1 (4.7)	0.397^3^
Smoking status				0.633^2^
Non-smoker (number (%))	52 (40)	11 (42)	41 (40)	
Former smoker (number (%))	66 (51)	14 (54)	52 (50)	
Current smoker (number (%))	11 (9)	1 (4)	10 (10)	
Taking cardiovascular medication (number (%))	90 (87)	19 (73)	71 (69)	0.813^2^
Taking vitamins (number (%))	25 (19)	2 (8)	23 (22)	0.104^2^
Taking low-dose aspirin (number (%))	41(32)	4 (15)	37 (36)	0.059^2^

SD: Standard deviation.

^1^Mann Whitney U test.

^2^Pearson’s chi-square test.

^3^Independent samples t-test.

**Table 2:** Percentage of leukocyte populations and cell ratios in nAMD patients and controls.

**Variables**	**Controls (mean** ± **SD) n*** *= **26**	**nAMD (mean** ± **SD) n*** *= **103**	**Univariate analysis**	**Multivariate analysis (age and gender)**			**Multivariate analysis (age, gender, cardiovascular disease and aspirin intake)**		
			***P*** **value nAMD vs Controls**^1^	***P*** **value nAMD vs Controls**^2^	**Odds ratio**	**95% confidence interval for odds ratio**	***P*** **value nAMD vs Controls**^3^	**Odds ratio**	**95% confidence interval for odds ratio**
Cell subsets (FSC/SSC plot)									
Lymphocytes (%)	30.45 ± 7.82	26.51 ± 6.97	**0.013**	**0.033**	0.94	0.88–0.99			
Monocytes (%)	7.28 ± 1.88	7.85 ± 2.58	0.292						
Neutrophils (%)	59.80 ± 8.73	63.71 ± 7.60	**0.025**	0.052	1.06	1.00–1.12			
Neutrophil/Lymphocyte ratio	2.16 ± 0.84	2.66 ± 1.08	**0.016**	**0.037**	17.56	1.19–258.21			
Cell subsets (CD antigens)									
CD14^+^ (%)	7.35 ± 1.95	7.61 ± 2.24	0.628						
CD4^+^ (%)	12.21 ± 3.92	11.46 ± 4.25	0.301						
CD8^+^ (%)	5.86 ± 2.96	5.06 ± 3.49	0.137						
CD19^+^ (%)	3.41 ± 2.16	2.78 ± 1.48	0.126						
CD56^+^ (%)	3.27 ± 1.66	3.46 ± 1.94	0.834						
CD11b^+^ (%)	67.40 ± 9.00	72.77 ± 7.68	**0.006**	**0.015**	1.08	1.01–1.14			
CD16^hi^HLA-DR^−^ Neutrophils (%)	57.80 ± 9.05	63.19 ± 7.87	**0.003**	**0.009**	1.07	1.02–1.13			
CD16^hi^HLA-DR^−^/(CD4 + CD8) ratio	3.51 ± 1.49	4.42 ± 1.88	**0.031**	0.072	10.24	0.81–129.24			
CD16^hi^HLA-DR^−^/CD4 ratio	5.34 ± 2.34	6.34 ± 2.74	0.100						
CD16^hi^HLA-DR^−^/CD8 ratio	13.80 ± 11.28	19.82 ± 16.22	0.051	0.072	3.97	0.88–17.88	**0.041**	5.66	1.07–29.79
CD16^hi^HLA-DR^−^/CD19 ratio	25.69 ± 21.26	32.14 ± 26.91	0.085						
CD16^hi^HLA-DR^−^/CD56 ratio	22.41 ± 11.25	25.75 ± 19.89	0.714						

**Bold** *P*<0.05; SD: Standard deviation.

^1^Independent samples t-test.

^2^Multivariable logistic regression; corrected for age and gender.

^3^Multivariable logistic regression; corrected for age, gender, cardiovascular disease and aspirin intake.

**Table 3:** Percentage of leukocyte populations and cell ratios in patients with nAMD with and without subretinal fibrosis.

**Variables**	**Controls (mean** ± **SD) n*** *= **26**	**Fibrosis absent (mean** ± **SD) n*** *= **66**	**Fibrosis present (mean** ± **SD) n*** *= **37**	**Univariate analysis**	**Multivariate analysis (age and gender)**		
				***P*** **value fibrosis absent vs present**^1^	***P*** **value fibrosis absent vs present**^2^	**Odds ratio**	**95% confidence interval for odds ratio**
Cell subsets (FSC/SSC plot)							
Lymphocytes (%)	30.45 ± 7.82	26.17 ± 7.4	27.11 ± 6.19	0.517			
Monocytes (%)	7.28 ± 1.88	8.09 ± 2.71	7.43 ± 2.32	0.215			
Neutrophils (%)	59.80 ± 8.73	63.80 ± 8.25	63.55 ± 6.37	0.869			
Neutrophil/Lymphocyte ratio	2.16 ± 0.84	2.75 ± 1.22	2.51 ± 0.77	0.515			
Cell subsets (CD antigens)							
CD14^+^ (%)	7.35 ± 1.95	7.75 ± 2.39	7.35 ± 1.96	0.447			
CD4^+^ (%)	12.21 ± 3.92	10.82 ± 4.08	12.44 ± 4.37	**0.042**	**0.026**	37.08	1.53–899.43
CD8^+^ (%)	5.86 ± 2.96	5.39 ± 3.8	4.51 ± 2.85	0.326			
CD19^+^ (%)	3.41 ± 2.16	2.95 ± 1.69	2.53 ± 1.07	0.249			
CD56^+^ (%)	3.27 ± 1.66	3.49 ± 1.57	3.42 ± 2.34	0.340			
CD11b^+^ (%)	67.40 ± 9.00	72.97 ± 8.47	72.48 ± 6.55	0.797			
CD16^hi^HLA-DR^−^ Neutrophils (%)	57.80 ± 9.05	63.33 ± 8.7	62.95 ± 6.21	0.798			
CD16^hi^HLA-DR^−^/(CD4 + CD8) ratio	3.51 ± 1.49	4.60 ± 2.06	4.13 ± 1.56	0.462			
CD16^hi^HLA-DR^−^/CD4 ratio	5.34 ± 2.34	6.80 ± 3.07	5.65 ± 1.97	0.120			
CD16^hi^HLA-DR^−^/CD8 ratio	13.80 ± 11.28	19.10 ± 15.52	21.02 ± 17.48	0.381			
CD16^hi^HLA-DR^−^/CD19 ratio	25.69 ± 21.26	31.16 ± 27.45	33.55 ± 26.44	0.354			
CD16^hi^HLA-DR^−^/CD56 ratio	22.41 ± 11.25	22.91 ± 15.72	29.25 ± 23.86	0.274			

**Bold** P < 0.05; SD: Standard deviation.

^1^Independent samples t-test.

^2^Multivariable logistic regression; corrected for age and gender.

**Table 4:** Percentage of leukocyte populations and cell ratios in nAMD patients with CNV and RAP.

				**Univariate analysis**			**Multivariate analysis (age and gender)**		
**Variables**	**Controls (mean** ± **SD) n*** *= **26**	**CNV (mean** ± **SD) n*** *= **66**	**RAP (mean** ± **SD) n*** *= **13**	***P*** **value CNV vs RAP**^1^	***P*** **value CNV vs RAP vs controls**^2^	***P*** **value Bonferroni** ***post hoc*** **test**	***P*** **value CNV vs RAP vs controls**^3^	**Odds ratio**	**95% confidence interval for odds ratio**
Cell subsets (FSC/SSC plot)									
Lymphocytes (%)	30.45 ± 7.82	27.13 ± 7.32	24.62 ± 6.36	0.254	**0.047**	0.161^4^ 0.064^5^			
Monocytes (%)	7.28 ± 1.88	7.84 ± 2.38	8.44 ± 4.02	0.468	0.382				
Neutrophils (%)	59.80 ± 8.73	63.28 ± 7.55	65.35 ± 8.01	0.373	0.075				
Neutrophil/Lymphocyte ratio	2.16 ± 0.84	2.62 ± 1.17	2.87 ± 0.96	0.329	0.055				
Cell subsets (CD antigens)									
CD14^+^ (%)	7.35 ± 1.95	7.61 ± 2.05	8.45 ± 3.57	0.336	0.472				
CD4^+^ (%)	12.21 ± 3.92	11.88 ± 4.75	10.77 ± 3.02	0.600	0.614				
CD8^+^ (%)	5.86 ± 2.96	5.25 ± 3.66	4.69 ± 2.49	0.978	0.425				
CD19^+^ (%)	3.41 ± 2.16	2.86 ± 1.37	2.19 ± 1.23	0.117	0.110				
CD56^+^ (%)	3.27 ± 1.66	3.50 ± 2.16	3.39 ± 1.71	0.801	0.958				
CD11b^+^ (%)	67.40 ± 9.00	72.00 ± 7.92	76.72 ± 5.42	0.082	**0.007**	0.086^4^ **0.009**^5^	**0.046**^4^ **0.033**^5^	1.07 1.15	1.00 ± 1.14 1.01 ± 1.30
CD16^hi^HLA-DR^−^ Neutrophils (%)	57.80 ± 9.05	62.92 ± 8.09	63.26 ± 7.77	0.888	**0.025**	**0.027**^4^ 0.166^5^	**0.015**^4^ 0.304^5^	1.08 1.05	1.01 ± 1.14 0.96 ± 1.15
CD16^hi^HLA-DR^−^/(CD4 + CD8) ratio	3.51 ± 1.49	4.25 ± 1.92	4.51 ± 1.56	0.487	0.150				
CD16^hi^HLA-DR^−^/CD4 ratio	5.34 ± 2.34	6.26 ± 2.94	6.40 ± 2.16	0.595	0.317				
CD16^hi^HLA-DR^−^/CD8 ratio	13.80 ± 11.28	20.40 ± 18.82	17.00 ± 8.09	0.997	0.221				
CD16^hi^HLA-DR^−^/CD19 ratio	25.69 ± 21.26	31.21 ± 27.79	45.60 ± 40.21	0.131	0.083				
CD16^hi^HLA-DR^−^/CD56 ratio	22.41 ± 11.25	27.33 ± 23.06	23.06 ± 10.91	0.942	0.922				

**Bold** *P *< 0.05; SD: Standard deviation.

^1^Independent samples t-test.

^2^One-way ANOVA.

^3^Multinomial logistic regression corrected for age and gender.

^4^Controls vs CNV.

^5^Controls vs RAP.
