# Supplementary material for: Alterations in Circulating Immune Cells in Neovascular Age-Related Macular Degeneration
Source: Sci Rep. 2015 Nov 17;5:16754. doi: 10.1038/srep16754 (PMC4648089; doi:10.1038/srep16754)
Supplement: Supplementary Information [file srep16754-s1.doc]

**Title** “Alterations in Circulating Immune Cells in Neovascular Age-Related Macular Degeneration”Judith **Authors**: Judith Lechner, Mei Chen, Ruth E. Hogg, Levente Toth, Giuliana Silvestri, Usha Chakravarthy and Heping Xu.


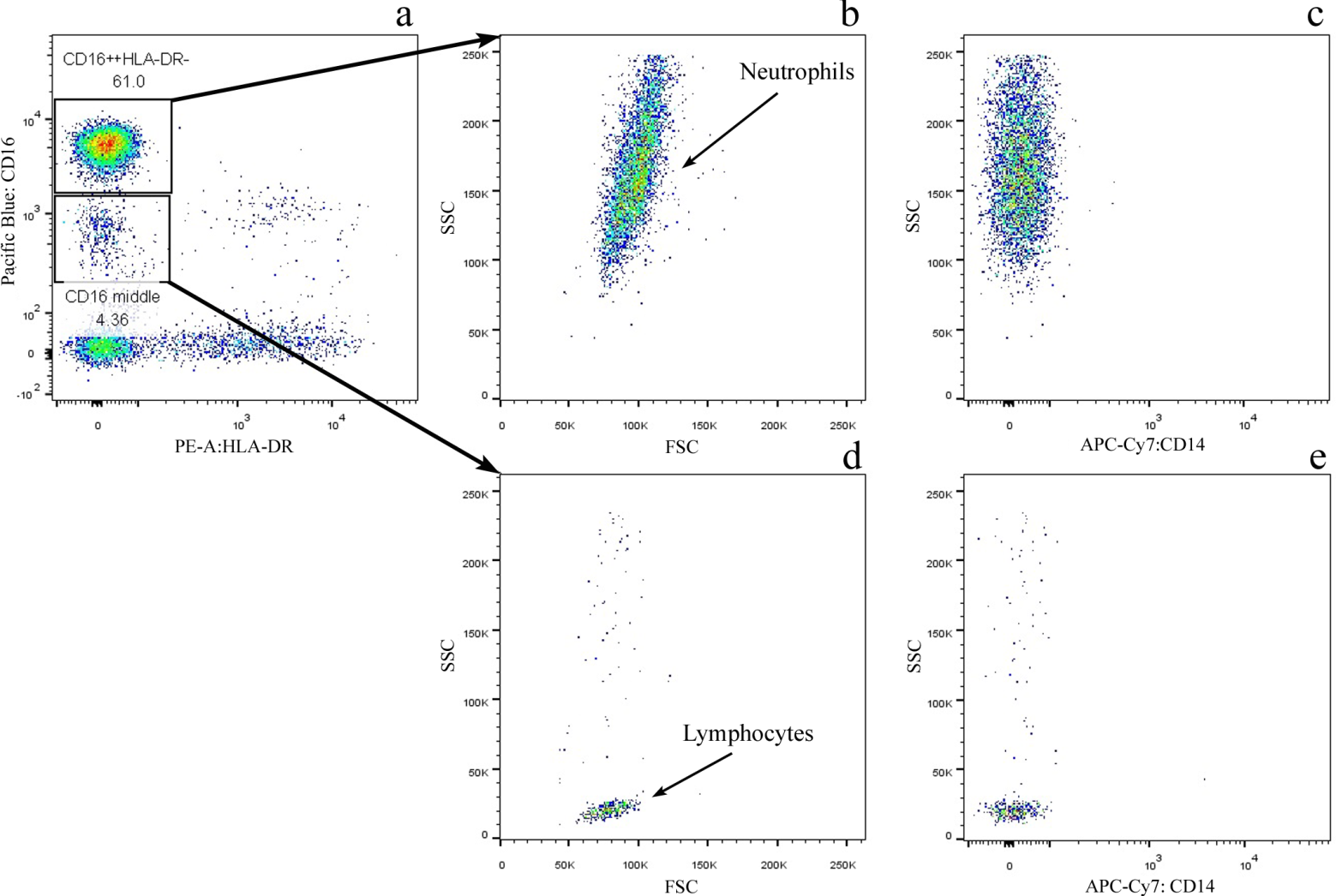


**Supplement figure S1: Phenotyping of CD16^+^HLA-DR^-^ cells**. (a) In the CD16 vs HLA-DR plot, two populations of CD16^+^HLA-DR^-^ cells can be identified: CD16^hi^HLA-DR^-^ and CD16^lo^HLA-DR^-^ cells. CD16^hi^HLA-DR^-^ cells had FSC/SSC profile of neutrophils (b) and were CD14^-^ (c). CD16^lo^HLA-DR^-^ cells had a FSC/SSC profile of lymphocytes (d) and were also CD14^-^ (e).

**Supplemental Table S1:** Percentage of leukocyte populations and cell ratios in nAMD patients not responding, partially responding and completely responding to anti-VEGF therapy

| **Variables** | **Controls (mean ± SD)**  **n = 26** | **Non responders (mean ± SD)**  **n = 3** | **Partial responders**  **(mean ± SD)**  **n = 52** | **Complete responders (mean ± SD)**  **n = 48** | ***P value***  **partial vs complete responders^1^** |
| --- | --- | --- | --- | --- | --- |
| **Cell subsets (FSC/SSC plot)** | | | | | |
| Lymphocytes (%) | 30.45 ± 7.82 | 26.07 ± 4.43 | 26.86 ± 7.65 | 26.15 ± 6.4 | 0.621 |
| Monocytes (%) | 7.28 ± 1.88 | 8.96 ± 1.66 | 7.78 ± 2.5 | 7.86 ± 2.74 | 0.886 |
| Neutrophils (%) | 59.80 ± 8.73 | 62.33 ± 5.84 | 63.60 ± 8.13 | 63.92 ± 7.2 | 0.833 |
| Neutrophil/Lymphocyte ratio | 2.160 ± 0.84 | 2.46 ± 0.6 | 2.68 ± 1.23 | 2.66 ± 0.94 | 0.800 |
| **Cell subsets (CD antigens)** | | | | | |
| CD14+ (%) | 7.35 ± 1.95 | 8.68 ± 1.76 | 7.55 ± 2.12 | 7.59 ± 2.41 | 0.939 |
| CD4+ (%) | 12.21 ± 3.92 | 11.67 ± 2.75 | 12.07 ± 4.44 | 10.80 ± 4.1 | 0.124 |
| CD8+ (%) | 5.86 ± 2.96 | 3.57 ± 2.54 | 4.86 ± 3.29 | 5.38 ± 3.75 | 0.392 |
| CD19+ (%) | 3.41 ± 2.16 | 3.41 ± 1.55 | 3.00 ± 1.64 | 2.44 ± 1.19 | 0.076 |
| CD56+ (%) | 3.27 ± 1.66 | 2.61 ± 0.76 | 3.46 ± 2.04 | 3.54 ± 1.9 | 0.618 |
| CD11b+ (%) | 67.40 ± 9.00 | 73.07 ± 4.37 | 72.28 ± 7.81 | 73.33 ± 7.93 | 0.589 |
| CD16^hi^HLA-DR^-^ Neutrophils (%) | 57.80 ± 9.05 | 61.77 ± 5.73 | 63.08 ± 8.22 | 63.39 ± 7.71 | 0.848 |
| CD16^hi^HLA-DR-/(CD4 + CD8) ratio | 3.51 ± 1.49 | 4.24 ± 1.35 | 4.23 ± 1.96 | 4.63 ± 1.84 | 0.308 |
| CD16^hi^HLA-DR-/CD4 ratio | 5.34 ± 2.34 | 5.55 ± 1.63 | 5.89 ± 2.43 | 6.89 ± 3.04 | 0.145 |
| CD16^hi^HLA-DR-/CD8 ratio | 13.80 ± 11.28 | 22.99 ± 13.07 | 21.07 ± 17.33 | 18.25 ± 15.26 | 0.465 |
| CD16^hi^HLA-DR-/CD19 ratio | 25.69 ± 21.26 | 21.12 ± 10.08 | 28.25 ± 18.24 | 37.82 ± 34.96 | 0.121 |
| CD16^hi^HLA-DR-/CD56 ratio | 22.41 ± 11.25 | 24.86 ± 6.01 | 27.32 ± 23.21 | 23.81 ± 15.89 | 0.743 |
| ^1^ Independent samples t-test  SD: Standard deviation | | | | | |
